# Modulation of Predictive Coding in Auditory Paradigms of Varying Complexity in Children With Developmental Language Disorder

**DOI:** 10.1111/ejn.70503

**Published:** 2026-04-24

**Authors:** Francisco J. Ruiz‐Martínez, Elena I. Rodríguez Martínez, Brenda Angulo Ruiz, Ana Gómez Trevióo, Raquel Muñoz Pradas, Sheyla Andalia, Carlos M. Gómez

**Affiliations:** ^1^ Department of Experimental Psychology University of Seville Seville Spain; ^2^ Victoria Eugenia Hospital Cruz Roja (UDIATE) Seville Spain; ^3^ Osuna University Sevilla Spain

**Keywords:** auditory design, children, DLD, ERPs, passive paradigm, predictive coding

## Abstract

Developmental language disorder (DLD) is a persistent difficulty in the acquisition and use of expressive and/or receptive language, which negatively impacts academic and social development. The present study evaluated the validity of the statistical learning model proposed to account for language difficulties in children with DLD. To this end, two auditory paradigms of varying complexity, framed within predictive coding theory, were passively presented to children diagnosed with DLD and to typically developing children without neurological impairments. The paradigms consisted of stimulus sequences with decreasing or increasing frequencies, interspersed with the sporadic occurrence of unexpected tone endings. The psychophysiological response was recorded using EEG, focusing on the P1, mismatch negativity (MMN), postimperative negative variation (PINV), and contingent negative variation (CNV) components. Results showed an absent MMN and a higher P1 response to deviant tones in children with DLD, suggesting an impaired development of frontal MMN generators, potentially compensated by activity in the primary auditory cortex. DLD participants also showed increased PINV and CNV responses during the most complex paradigm, which could imply greater cognitive effort and resource allocation for reassessment of stimulus patterns. Finally, incomplete maturation of frontal areas in children within this age range (3–11 years) was proposed as a possible explanation for the absence of differences between groups in P1 and N1/MMN responses elicited by simple and complex conditions. These findings support statistical learning as a valid model for understanding the possible neural basis of DLD and highlight this predictive EEG design as a potential protocol for early detection.

## Graphical abstract

Event‐related potentials elicited in children with specific language impairment (SLI) and typically developing (controls), averaged across the F3, F4, Fz, C3, C4, and Cz electrodes. The upper panel displays the comparison between first‐ and second‐order complexity paradigms, averaging standard and deviant trials, with the baseline established previous to the first stimulus of the sequence (S1). The lower panel shows the standard and deviant comparison, averaging both paradigms, with the baseline before the last stimulus of the sequence (S4).

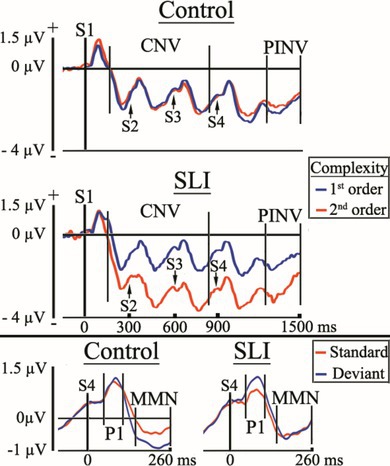

**Abbreviations**
CNV: contingent negative variation
D: deviant
DLD: developmental language disorder
MMN: mismatch negativity
ND: normodevelopment
PINV: postimperative negative variation
S: standard

## 1 Introduction

Predictive coding is a theory of established scientific interest that proposes that the brain continuously extracts patterns from the environment in order to construct generative models based on prior experience. The objective of this cognitive function would be to anticipate future events while minimizing prediction error, thereby guiding attention and behaviour (Friston 2005; Winkler and Czigler 2012; Heilbron and Chait 2018).

Under this framework, prediction error is considered the neural mechanism involved in detecting the mismatch between a prediction, based on previously perceived stimulation, and incoming sensory input. This discrepancy elicits a psychophysiological response whose magnitude is proportional to the degree of deviation, allowing the predictive model to be updated and improve future predictions (Friston and Kiebel 2009; Friston 2010; Hohwy 2012; Brodski et al. 2015). In line with this, a broadly supported hypothesis proposes a Bayesian probabilistic model to explain the way in which the brain generates these predictions and updates them to reduce prediction error (Knill and Pouget 2004; Doya et al. 2007; Gómez and Flores 2011; Friston et al. 2017; Gómez et al. 2019).

The psychophysiological functioning of predictive coding and its potential impairment in clinical disorders have been extensively investigated using EEG (González‐Gadea et al. 2015; Fogelson and Diaz‐Brage 2021; Qela et al. 2025). For this purpose, neurobiological markers sensitive to psychophysiological impairments in clinical populations, such as the auditory P1 (Schall et al. 1997; Ruiz‐Martínez et al. 2020; Sharma et al. 2005), the auditory mismatch negativity (MMN) (Näätänen et al. 2014; Erickson et al. 2016; Tada et al. 2019), the contingent negative variation (CNV) (Osborne et al. 2020; Lahoz et al. 2023; Prata et al. 2023), and the postimperative negative variation (PINV) (Klein et al. 1996; Diener et al. 2009; Werner et al. 2011) have been widely examined.

The auditory P1 is an early positive event‐related potential (ERP) component, typically elicited approximately 60–100 ms post‐stimulus, and is generated in the primary and secondary auditory cortices (Muñoz‐Caracuel et al. 2021). Additionally, it may also exhibit frontal activation during tasks with higher attentional or cognitive demands, as a consequence of the involvement of thalamocortical projections. This potential is considered a reflection of initial and automatic sound detection, although it has been proposed to be modulated by active attention (Picton et al. 1974; Celesia 1976; Alho et al. 1994; Herrmann and Knight 2001; Ponton and Eggermont 2001; Martin et al. 2008; Giuliano et al. 2014). The auditory P1 is not commonly evaluated in predictive paradigms due to its early elicitation, which generally precedes cognitive processes such as those involved in predictive coding. However, in some studies, the P1 and even earlier positive components have been shown to be sensitive to stimulus omission or deviations in temporal stimulus onset (Bendixen et al. 2009; Schwartze et al. 2013; Lee et al. 2024).

The MMN is the most important neural marker of preattentive auditory discrimination. According to predictive coding theory, it reflects the prediction error generated by the violation of the expectation formed by a repetitive stimulus pattern (standard), due to the occurrence of an unexpected stimulus that changes the pattern (deviant) (Winkler 2007; Garrido et al. 2009; Wacongne et al. 2011). This negative component in healthy young adults typically peaks between 150 and 250 ms following change onset and presents a predominantly frontocentral topography (Näätänen et al. 2007). It can be generated passively (without attention to the stimulation) and is modulated by attentional processes (Näätänen and Alho 1995; Sussman et al. 2003; Näätänen et al. 2012). Although it is widely associated with prediction error, the discussion about whether this component is a modulation of the N1 caused by fresh afferent activity in cortical neurons undergoing adaptation or habituation processes remains ongoing (May and Tiitinen 2010; O'Reilly and O'Reilly 2021).

The CNV is a slow negative brain potential commonly elicited by stimulus anticipatory paradigms, as it is fundamentally generated by the expectancy that a warning signal produces regarding the occurrence of a target stimulus (Walter et al. 1964; Brunia et al. 2011; Gómez et al. 2019; Kóbor et al. 2021). It is considered a reflection of cortical arousal and attention generated by response anticipation, mainly in motor tasks (Kropp et al. 2001; Kononowicz and Penney 2016; Fattapposta et al. 2024), although it has also been suggested in passive studies to reflect sensory expectations (Mento 2013; Ruiz‐Martínez et al. 2022; Muñoz‐Caracuel et al. 2024).

In some experimental settings, the CNV recovery to baseline after target onset is sustained over time, forming the so‐called PINV component (Delaunoy et al. 1978; Kathmann et al. 1990). This component, usually associated with paradigms designed to induce a perceived lack of control over aversive stimuli, uncertainty regarding response correctness, or the reassessment of ambiguous contingencies (Rockstroh et al. 1979; Kathmann et al. 1990; Klein et al. 1996), has recently been elicited in adults by our research group using the same experimental protocol, suggesting a role of the PINV in predictive coding (Ruiz‐Martínez et al. 2022). Both the CNV and PINV topographies are predominantly located in the frontal region, although the CNV is also generated in central and parietal areas (Cui et al. 2000; Gómez et al. 2001; Bender et al. 2006; Diener et al. 2009).

These components will be analyzed in the present research in a sample of children with developmental language disorder (DLD) and typically developing (ND) children, through a predictive coding approach, with the objective of studying potential alterations in statistical learning related to language.

As described in the DSM‐5 (American Psychiatric Association [APA] 2013), DLD is included within the broader category of DLDs, consisting of persistent difficulties in the acquisition and use of expressive and/or receptive language. To be considered DLD, these limitations must emerge in early development within adequate social environments and in the absence of sensory, emotional, physical, or cognitive deficits (Villegas 2022; APA 2013). As a consequence of these language difficulties, individuals with DLD often present delayed or impaired reading and/or academic development (Young et al. 2002; Pennington and Bishop 2009), which can negatively affect adolescence across multiple domains (social, emotional, behavioral, etc.) (Wadman, Botting, et al. 2011; Wadman et al. 2011a, 2011b), and may lead to reduced well‐being and life satisfaction in adulthood (Arkkila et al. 2008).

The genetic involvement in DLD has been extensively studied (Bishop 2002, 2009, 2013; Li and Bartlett 2012), and many theoretical models have been suggested to explore its underlying mechanisms. Among the most relevant hypotheses is the temporal processing hypothesis of language, which proposes a desynchronization between neural activity and speech rhythms present in prosodic and phonological expression (Goswami 2019). Another significant theory is the speed‐of‐processing limitation hypothesis, which proposes a psychophysiological difficulty in distinguishing phonemes with short temporal transitions due to slower auditory processing (Tallal 2004). Finally, the statistical learning model also stands out, suggesting a reduced ability to extract implicit language patterns in individuals with this disorder (Lammertink et al. 2017). This last theory is the approach followed in the present study and could underlie the DLD limitations in acquiring grammatical structures.

More specifically, the statistical learning model proposes that language is structured by phonological and syntactic dependencies that establish hierarchical links between words, where certain elements (dependents) are governed by others (heads), thus defining the distribution of grammatical and semantic roles across sentences. This model would suggest that DLD could present a deficit in the statistical processing necessary for establishing dependencies in adjacent and, primarily, nonadjacent elements within sentences, through sequential and probabilistic pattern extraction (Saffran et al. 1996; Saffran 2002; Gibson 1998; Hsu et al. 2014).

Several psychophysiological studies have analyzed DLD using ERPs (Bishop and McArthur 2005), suggesting that speech processing deficits and differences in the spatial distribution of attentional auditory resources are associated with a delayed P1 component (Shafer et al. 2007; An et al. 2022). A relationship has also been found between a reduced or absent MMN and attentional deficits in auditory stimulus discrimination (Shafer et al. 2005; Datta et al. 2010; Kujala and Leminen 2017). A reduction in the late positive component (LPC), which is involved in postlexical reanalysis and integration, has also been reported (Haebig et al. 2018). A delayed or absent N400, commonly interpreted as a semantic index of prediction error, has likewise been observed in individuals with DLD (Shafer et al. 2005; Sabisch et al. 2006; Haebig et al. 2018). Similarly, the absence or reduction of the early left‐anterior negativity (ELAN) and the P600 may be related to deficits associated with DLD in the early detection of syntactic violations and in reanalysis or repair processes, respectively (Fonteneau and van der Lely 2008; Royle and Courteau 2014; Purdy et al. 2014; Haebig et al. 2017).

N400 and P600 components have been widely studied within the framework of Bayesian processing to evaluate the statistical learning model of language development, often using sentences with unexpected or incongruent elements (e.g., the cool water burned him) (Kutas and Hillyard 1984; Herten et al. 2005; León‐Cabrera et al. 2017; Wang et al. 2023; Michaelov et al. 2024). However, to our knowledge, no ERP study to date has investigated DLD within a purely predictive paradigm. This research gap motivated the present study, along with the need for an early diagnostic protocol to identify DLD before language emergence, enabling timely interventions to reduce cognitive and social deficits. To this end, the P1, MMN, CNV, and PINV components were evaluated to detect potential alterations in DLD through the presentation of two passive auditory conditions of different complexity in children with this disorder and ND children. These components have been previously obtained and analyzed by our group during passive presentation of auditory stimuli of tone sequences (Ruiz‐Martínez et al. 2022; Muñoz‐Caracuel et al. 2024). The tone sequences were constructed to increase or decrease the predictability of the last tone frequency of the sequence in first‐order complexity (structure of the tone sequences: ABCD, ABCD, ABC[F]; brackets indicate a nonpredictable ending of the sequence, or deviant trial as opposed to the two previous predictable or standard trials) and in second‐order complexity (the exact tones vary but maintain the same regularity relationships: ABCD, EFGH, IJK[M], with the last sequence being the deviant trial compared with the two considered as standard trials). P1, N1/MMN, PINV, and CNV were obtained for both first‐order and second‐order complexity deviant trials.

The following three hypotheses were proposed to explain how a potential deficit in statistical learning might influence the psychophysiological responses of DLD participants compared with the control group:

1. ND children were expected to show a higher amplitude for the deviant than for standard trials, primarily at the MMN latency (where this effect is most commonly observed) and secondarily also at the PINV. Conversely, the MMN amplitude for deviants would be attenuated in DLD participants.
2. A greater response to the first‐order condition (high repetition of the same tones in the auditory sequences), compared with the second‐order condition (low repetition of tone sequences), was expected for the P1 and MMN components in both groups. The direction of this effect would indicate the degree of maturation of predictive mechanisms and consequently the prediction error. Specifically, a higher response to the second‐order condition would suggest a more adult‐like maturation pattern, reflecting the recruitment of additional neural resources to extract more complex patterns. Conversely, a higher amplitude in the first‐order condition would suggest incomplete development of these brain areas.
3. A higher amplitude was expected for the second‐order condition in both the CNV and PINV, primarily in the DLD group. The increase in CNV amplitude would reflect an enhanced preparatory process associated with greater cognitive effort, whereas the increase in PINV amplitude would reflect a heightened allocation of resources for stimulus pattern reassessment.

If confirmed, these hypotheses would support the early use of this passive EEG paradigm for diagnosing DLD in children, thanks to its noninvasive and child‐friendly nature. This is especially relevant given the high prevalence of DLD, which affects between 1.4% and 16.2% of school‐aged children worldwide (Tomblin et al. 1997; Villegas 2022).

## 2 Materials and Methods

### 2.1 Participants

A total of 86 subjects divided into a normodevelopmental (ND) group and a clinical group diagnosed with DLD were recorded in two experimental conditions of different complexity. The ND group was composed of 51 participants aged 3–11 years (19 males and 32 females, mean age in days = 2723.94 ± 625.03 SD, range 1739–4023 days old) and the clinical group of 35 participants aged 3–10 years (25 males and 10 females, mean age in days = 2484.49 ± 589.15 SD, range 1271–3916 days old). We applied the Mann–Whitney U test, obtaining *p* = 0.079, which indicates that no significant differences were found between the age groups. Similarly, the Kolmogorov–Smirnov test showed *p* = 0.078, indicating no significant differences between the distributions of both groups. Due to excessive EEG artifacts in the recording of the complex condition, one subject from the clinical group was excluded from the analysis. Thus, the analyzed group in this condition was composed of 34 subjects aged 4–10 years (25 males and 9 females, mean age in days = 2520.18 ± 558.28 SD, range 1686–3916 days old).

The clinical group was recruited from the Unidad de Desarrollo Infantil y Atención Temprana (UDIATE: https://hospitalveugenia.com/udiate‐atencion‐temprana‐desarrollo‐infantil/) affiliated with the Hospital Victoria Eugenia, operating within the Spanish Red Cross, which is dedicated to the diagnosis and intervention of neurodevelopmental disorders in children. Inclusion in the study required a clinical report confirming language deficits, endorsed by language specialists from the clinical center. This confirmation was based on standardized assessments such as the Navarre Oral Language Test—Revised (PLON‐R; Aguinaga Ayerra 2005), the Illinois Test of Psycholinguistic Abilities (ITPA; Kirk and McCarthy 1961), the Clinical Evaluation of Language Fundamentals—Fifth Edition (CELF‐5; Wiig et al. 2013), the Kaufman Brief Intelligence Test (KBIT; Kaufman and Kaufman 2004), the Peabody Picture Vocabulary Test—Fifth Edition (PPVT‐5; Dunn 1959), or a structured clinical interview conducted by language therapists in accordance with DSM‐5 or ICD‐10 criteria. Before enrolment, otorhinolaryngologists confirmed normal auditory function using audiometric tests and, when needed, brainstem auditory evoked potentials, thereby ruling out hearing impairment as a confounding factor.

ND participants were recruited from various schools in Seville, Spain. Parental reports confirmed the absence of learning difficulties, neurological disorders, or developmental delays in these children.

Nonverbal cognitive abilities were assessed using the Kaufman Brief Intelligence Test (KBIT) for 80 participants (as some participants were unable to complete the test due to assessment constraints). The raw KBIT scores met the criteria for normal distribution and homogeneity of variances. Statistical comparison of the raw scores (RS) did not reveal significant differences in cognitive performance between the ND group (M = 23.79, SD = ±6.03) and the clinical group (M = 22.41, SD = ±6.94), as indicated by a *t* test (*t*(78) = 0.95, *p* = 0.35, *d* = 0.22).

Experiments were conducted with written informed consent from each participant and/or their parents or caregivers, following the Helsinki Protocol (code: 0818‐N‐21). The study was approved by the Biomedical Ethics Committee of the Junta de Andalucía.

### 2.2 Experimental Procedure

Auditory stimuli were delivered via two Dell A215 speakers positioned bilaterally at the participant's head level, at an intensity of 65 dB, measured using a Velleman DVM1326 sound level meter. Each auditory stimulus consisted of a 200‐ms sinusoidal tone with 20‐ms rise and fall times. Tones were generated using an online tone generator (https://www.wavtones.com/functiongenerator.php, last accessed April 15, 2019) and subsequently edited in Audacity 2.3.2.

**FIGURE 1:**
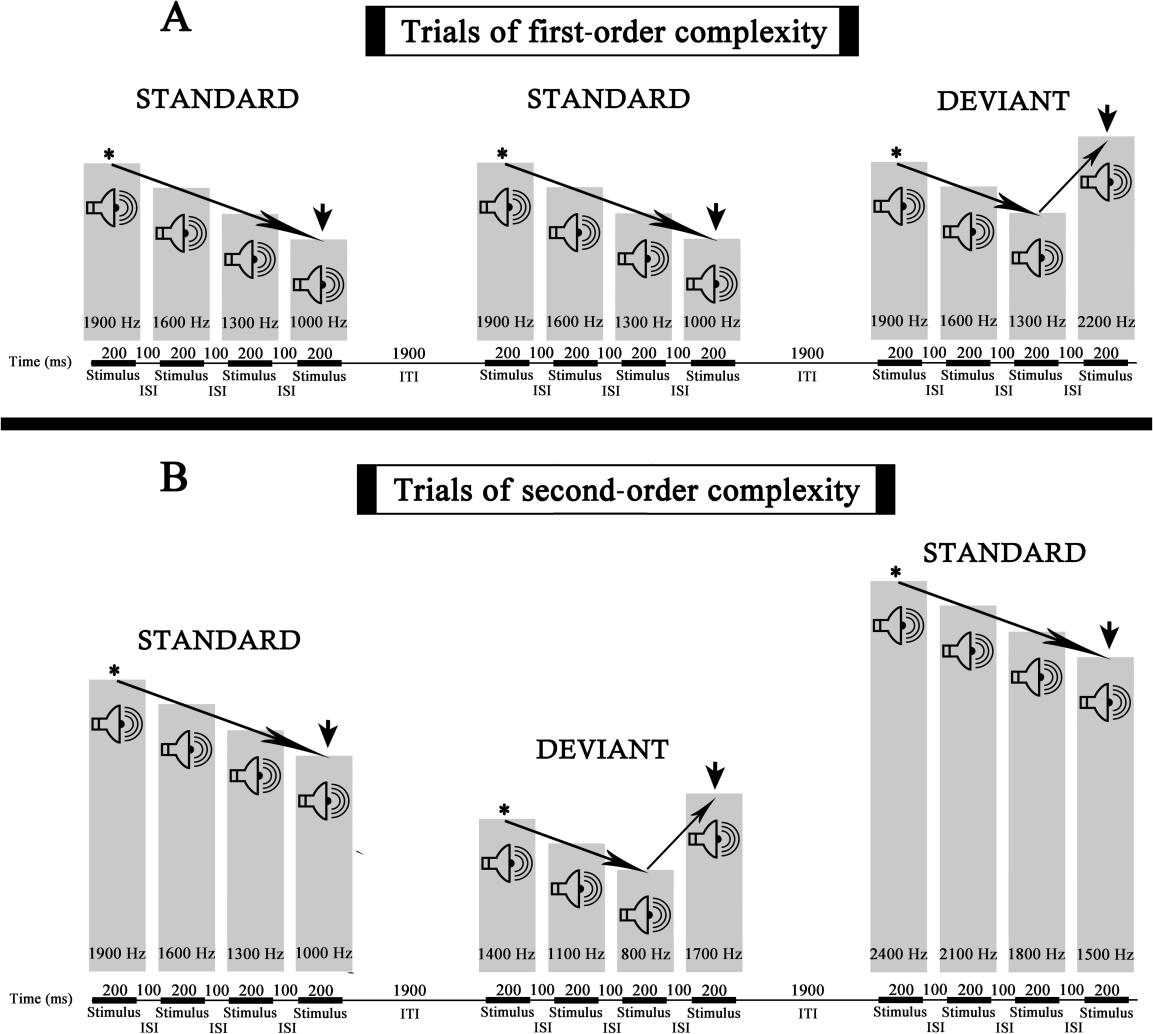
Experimental conditions. (A) The timeline below the bar graph shows the duration of the stimuli, the interstimulus intervals (ISIs), and the intertrial intervals (ITIs), and the bar graphs present the order and frequencies of the stimuli displayed in the standard and deviant descending sequences for the first‐order complexity. The fourth stimulus (S4), marked by arrows, defined the onset for time‐locking P1 and late N1 epochs. Asterisks indicate that the baseline for obtaining the CNV and PINV components was located just before the first stimulus (S1). (B) The same for the second‐order complexity condition.

The experimental conditions were programmed in E‐Prime 2.0 and included two conditions differing in sequence complexity: first‐order and second‐order. Each trial comprised a sequence of four tones (S1–S4; see Figure 1). To control for neural activation effects in the auditory cortex, sequences with increasing and decreasing frequencies were counterbalanced across participants.

Table 1 summarizes the frequencies used in both complexity conditions for ascending and descending sequences. In the first‐order condition, each trial type (standard: S and deviant: D; ascending and descending) was defined by a single, fixed stimulus sequence, allowing participants to form stable representations of the physical characteristics of the tones.

**TABLE 1:** Stimulus frequencies by condition. The table displays the frequencies (in Hz) of each stimulus sequence presented in the ascending and descending sound designs, across both complexity conditions (first‐ and second‐order) and trial types (standard and deviant).

Ascending		Descending	
Standard	Deviant	Standard	Deviant
**First‐order complexity**			
1300, 1600, 1900, 2200	1300, 1600, 1900, 1000	1900, 1600, 1300, 1000	1900, 1600, 1300, 2200
**Second‐order complexity**			
2800, 3100, 3400, 3700	2800, 3100, 3400, 2500	3400, 3100, 2800, 2500	3400, 3100, 2800, 3700
2300, 2600, 2900, 3200	2300, 2600, 2900, 2000	2900, 2600, 2300, 2000	2900, 2600, 2300, 3200
1800, 2100, 2400, 2700	1800, 2100, 2400, 1500	2400, 2100, 1800, 1500	2400, 2100, 1800, 2700
1300, 1600, 1900, 2200	1300, 1600, 1900, 1000	1900, 1600, 1300, 1000	1900, 1600, 1300, 2200
800, 1100, 1400, 1700	800, 1100, 1400, 500	1400, 1100, 800, 500	1400, 1100, 800, 1700

In contrast, the second‐order condition involved five distinct stimulus sequences per trial type, with a consistent frequency interval of 300 Hz between tones. Through this second‐order condition, we examined whether participants could detect the underlying frequency pattern while reducing the possibility of memorizing individual tone characteristics.

All trials had a total duration of 3 s, comprising four 200‐ms tones, three 100‐ms interstimulus intervals, and a 1900‐ms intertrial interval (Figure 1). Each participant completed a total of 604 trials: 241 S and 61 D trials per complexity condition, maintaining a 4:1 S‐to‐D ratio. The total experiment duration per participant was 30 min and 20 s.

The experimental blocks (first‐ and second‐order) were presented in counterbalanced order across participants, and trials were arranged considering the previous trial (SS, SD, DS, DD) with respective proportions of 59.1%, 15.7%, 15.7%, and 9.5% to ensure an adequate number of DD trials. The sequence of dyads was pseudorandomized such that at least one SS dyad followed any dyad containing a D trial, thus preserving the integrity of the S effect throughout the session.

### 2.3 EEG Recording

Signals were acquired from 20 scalp electrodes arranged according to the international 10–20 system (Fp1, Fp2, F7, F3, Fz, F4, F8, T3, C3, Cz, C4, T4, T5, P3, Pz, P4, T6, O1, O2), including a ground electrode. Given the potential sensory sensitivities in our participant population, we opted not to use ocular or mastoid electrodes. This decision facilitates replication across similar clinical groups and improves the quality of recordings by increasing the comfort of younger participants. A common reference was applied, and activity from electrodes Fp1, Fp2, F7, and F8 was used for the detection and removal of blink, and eye‐movement artifacts.

Participants were seated comfortably between the loudspeakers and instructed to remain relaxed while viewing a silent movie displayed on a laptop screen throughout the recording session.

Data acquisition was performed in direct current mode at a sampling rate of 1024 Hz, using a commercial analog‐to‐digital acquisition and analysis system (ANT, The Netherlands) with an amplification gain of 20,000. Electrode impedance was kept below 10 kΩ, and data were filtered offline.

### 2.4 Data Analysis

EEG recordings were analyzed using EEGLAB version 14.1.1 (Delorme and Makeig 2004), and Matlab 2023b (Mathworks Inc. 2023) scripts that were developed in‐house. A high‐pass filter of 0.05 Hz and a low‐pass filter of 45 Hz were applied to the EEG signal, in addition to the application of an artifact subspace reconstruction (ASR) algorithm, implemented via the EEGLAB function “clean_rawdata,” which was employed to correct data segments whose standard deviation (SD) exceeded 20 times the calibration data (Mullen et al. 2015).

We performed independent component analysis (ICA) to identify and remove artifacts including those from electronic sources, muscle activity, cardiac signals, blinks, and eye movements in the epoched data. These components were removed and the EEG signal was reconstructed.

The retained ICA components for the ND group were 12.63 ± 1.41 SD, range 9–16 (for the first‐order complexity) and 12.59 ± 1.15 SD, range 11–16 (for the second‐order complexity), whereas for the clinical group they were 12.49 ± 1.65 SD, range 10–16, and 12.59 ± 1.89 SD, range 9–17, respectively.

**FIGURE 2:**
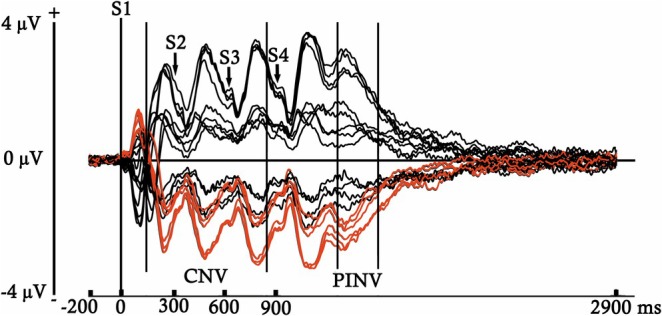
ERP averages obtained for both trial types (S and D), complexity conditions (first‐ and second‐order), and participant groups, computed for each electrode across the entire time window analyzed, using a 200 ms pre‐S1 onset baseline. In red, are marked the waveforms elicited by the electrodes selected for analysis (F3, F4, Fz, C3, C4, and Cz). Between bars appear the latencies selected to analyze the CNV and PINV components.

**FIGURE 3:**
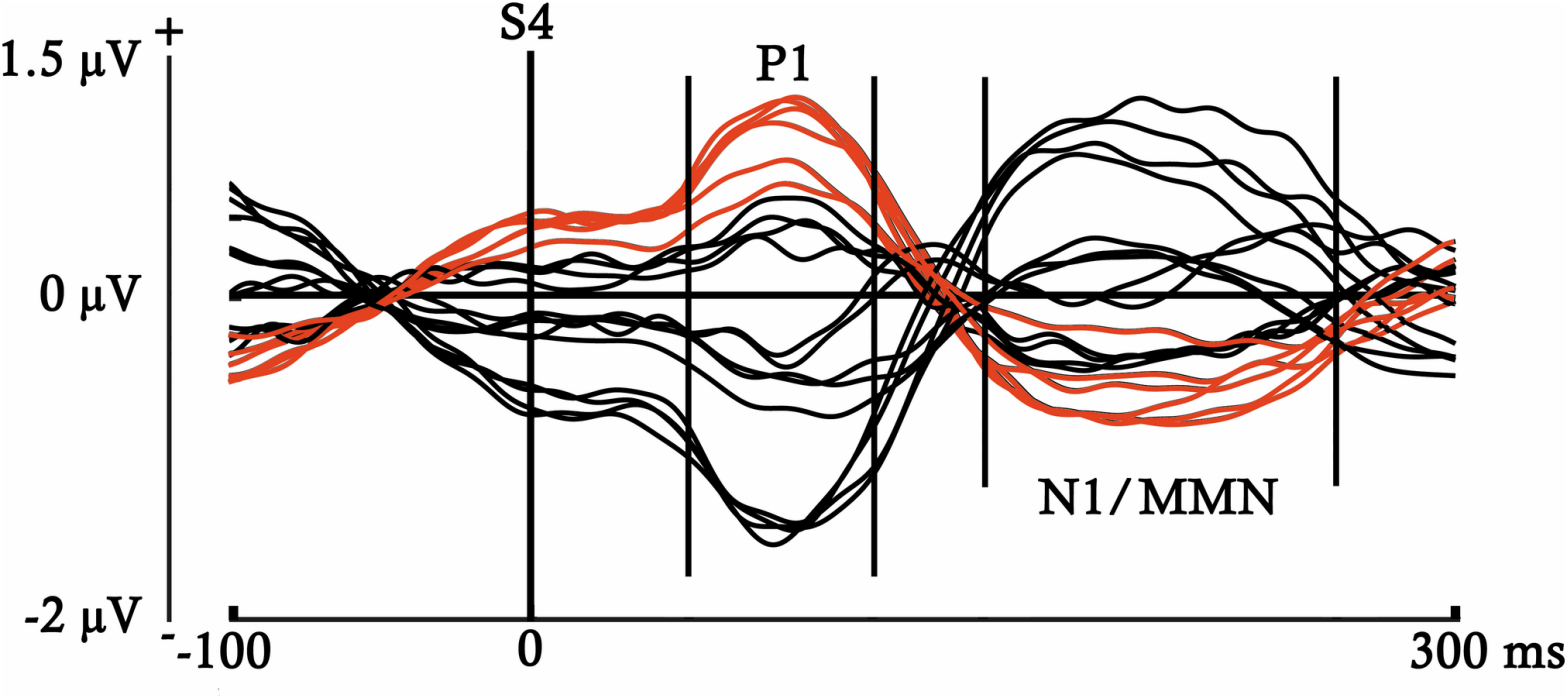
ERP averages obtained for both trial types (S and D), complexity conditions (first‐ and second‐order), and participant groups, computed for each electrode from S4 onset to 300 ms, using a 100 ms pre‐S4 onset baseline. In red, are marked the waveforms elicited by the electrodes selected for analysis (F3, F4, Fz, C3, C4, and Cz). Between bars appear the latencies selected to analyze the P1 and MMN components. The N1/MMN label indicates that the MMN was embedded within a latency similar to that of the N1 component.

Epochs were segmented using ERPLAB into different time windows, depending on the ERP component selected for analysis. This resulted in different averages for each participant, trial type (S or D), and complexity condition (first‐ or second‐order complexity). Grand‐average ERP images (Figures 2 and 3) were generated to identify the ERP components and to determine their respective latencies without introducing any condition or group bias. These figures present the activity recorded at each analyzed electrode across both participant groups and experimental conditions in two different time windows.

Figure 2 displays the entire interstimulus interval (0‐ to 2900‐ms post‐S1), using a baseline of 200 ms prior to S1, in order to identify components with longer temporal duration. In contrast, Figure 3 shows the voltage elicited during a shorter period following the onset of S4 (0‐ to 300‐ms post‐S4), with a baseline of 100 ms prior to S4, aiming to detect early components with shorter durations.

An artifact rejection voltage limit of ±130 mV was applied to the EEG in both the epochs selected to display Figures 2 (from 0‐ to 2900‐ms post‐S1) and 3 (from 900‐ to 1200‐ms post‐S1), and in all the epochs analyzed in this study (described below). This voltage rejection threshold is higher than that typically used in adults due to the higher spontaneous EEG amplitude observed in the pediatric population. This factor could lead to an excessively high number of rejected trials, potentially impairing the statistical power required to ensure optimal data quality. In this manner, all the epochs that exceeded ±130 mV in the time windows selected for each component, in any channel, complexity condition, and type of trial, were rejected (Tables 2 and 3).

**TABLE 2:** Accepted trials after voltage artifact rejection (first‐order condition). The table presents the mean, standard deviation, and range of accepted trials applying an artifact rejection threshold of ±130 mV, for both participant groups and trial types, within the first‐order complexity condition. Data are reported for four analyzed time windows: 0–2900 ms (used for plotting the ERP grand‐average); 150–850 ms (selected for the CNV component); 900–1200 ms (chosen for both the P1 and the MMN components); and 1200–1500 ms (designated for the PINV component). The mean and standard deviation (SD) were measured in microvolts (μV).

	First‐order complexity											
	ND group						DLD group					
	Standard			Deviant			Standard			Deviant		
Latency	Mean	SD	Range	Mean	SD	Range	Mean	SD	Range	Mean	SD	Range
0–2900 ms	233.73	±20.63	106–241	59.32	±4.88	32–61	225.42	±40.54	23–241	57.61	±9.18	11–61
150–850 ms	236.35	±18.62	108–241	60.04	±4.09	32–61	232.17	±31.38	56–241	59.17	±7.43	17–61
900–1200 ms	237.06	±18.36	109–241	60.18	±4.09	32–61	234.29	±28.52	74–241	59.56	±6.35	24–61
1200–1500 ms	236.86	±18.39	109–241	60.16	±4.08	32–61	232.89	±31.9	54–61	59.4	±6.77	21–61

**TABLE 3:** Accepted trials after voltage artifact rejection (second‐order condition). The table presents the mean, standard deviation, and range of accepted trials applying an artifact rejection threshold of ±130 mV, for both participant groups and trial types, within the second‐order complexity condition. Data are reported for four analyzed time windows: 0–2900 ms (used for plotting the ERP grand‐average); 150–850 ms (selected for the CNV component); 900–1200 ms (chosen for both the P1 and the MMN components); and 1200–1500 ms (designated for the PINV component). The mean and standard deviation (SD) were measured in microvolts (μV).

	Second‐order complexity											
	ND group						DLD group					
	Standard			Deviant			Standard			Deviant		
Latency	Mean	SD	Range	Mean	SD	Range	Mean	SD	Range	Mean	SD	Range
0–2900 ms	235.96	±10.17	172–241	59.71	±2.54	44–61	160.85	±69.59	18–240	42.09	±16.21	9–61
150–850 ms	238.88	±2.77	222–241	60.43	±1.59	50–61	208.32	±36.55	106–241	53.67	±8.3	26–61
900–1200 ms	239.67	±1.16	233–241	60.69	±0.62	58–61	227.65	±16.55	180–241	58.06	±4.03	46–61
1200–1500 ms	239.31	±2.93	220–241	60.51	±1.17	54–61	202.89	±8.86	98–241	51.82	±8.86	27–61

**FIGURE 4:**
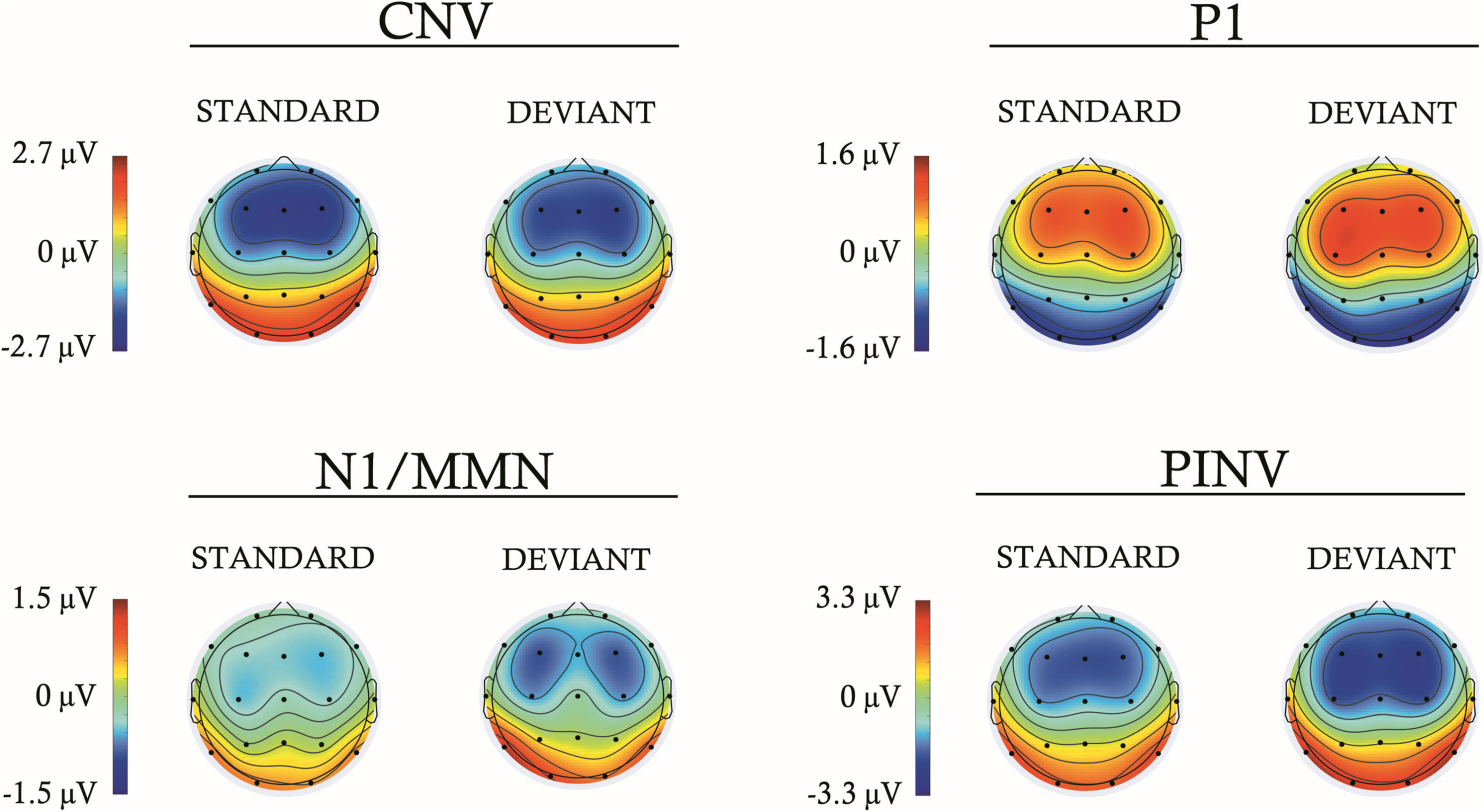
Topographies of the standard and deviant trials computed as the average of both complexity conditions (first‐ and second‐order) and participant groups (ND and DLD), in each component analyzed (CNV, P1, N1/MMN, and PINV). The N1/MMN label indicates that the MMN was embedded within a latency similar to that of the N1 component.

The topographies of the mean ERPs for both participant groups and complexity conditions, for S and D trials, are plotted in Figure 4 for the time windows selected for each component analyzed (described above) to determine the electrodes most relevant for the analysis.

The electrodes selected for analysis were F3, F4, Fz, C3, C4, and Cz, based on the larger amplitudes observed in Figures 2 and 3 (collapsed across conditions, groups, and subjects) and on our previous experience with the present experimental paradigm (Ruiz‐Martínez et al. 2022; Muñoz‐Caracuel et al. 2024). This selection is also supported by the well‐established involvement of frontocentral regions in the ERP components examined in the present study, as reported in the literature (Sharma et al. 2005; Elbert et al. 1982; Gómez et al. 2019; Näätänen et al. 2012). Although an inverted polarity relative to that observed at frontal electrodes was recorded at posterior electrodes, this may reflect dipolar sources located in the inferior bank of the lateral fissure within the auditory cortex. However, these more distant regions are likely to integrate nonauditory information and are generally not included in the analysis of auditory ERPs (Woldorff and Hillyard 1991; Näätänen et al. 2007; May and Tiitinen 2010; Paavilainen 2013). Importantly, based on our prior work with this paradigm, the ERP components of interest were defined a priori (CNV, P1, N1/MMN, and PINV) (Ruiz‐Martínez et al. 2022; Muñoz‐Caracuel et al. 2024).

A slow negativity from 150‐ms post‐S1 to the S4 onset was selected for analysis (see Figure 2), with a time window from 150‐ to 850‐ms post‐S1 and a baseline of 200 ms. This component would be interpreted as a CNV, suggesting that it has been elicited by the expectancy generated by the S1 (considered as the warning signal) about the appearance of the S4 (considered as the target) (Kononowicz and Penney 2016; Arjona Valladares et al. 2017). A progressive return to baseline was observed approximately from 350‐ms post‐S4 to the end of the inter‐stimulus interval (see Figure 2). This component would be interpreted as a PINV, as it appears to reflect the ERP recovery of the negativity generated by the CNV (Wagner et al. 1996; Ruiz‐Martínez et al. 2022). The time window was established from 345‐ to 600‐ms post‐S4 because the effect was more clearly observed within this latency. A baseline of 200 ms pre‐S1 was used due to the previously commented relationship of this component with the CNV.

An early and anterior positivity followed by a later and anterior negativity, possibly indexing the P1 and N1/MMN components, respectively, can be observed in Figure 3 after the S4 onset. The time windows selected for analysis were from 50‐ to 110‐ms post‐S4 in the case of the P1, and from 145‐ to 260‐ms post‐S4 for the MMN. For both components, the baseline was established 100 ms prior to the S4. Note that in the figures displayed, the MMN latency was labeled as N1/MMN. The reason is that, although this time window was selected to analyze the MMN, presenting both trial types separately would correspond to the N1 component, theoretically, since the MMN is obtained by subtracting the S from the D. As a clarification for the results and discussion sections, when we refer to ERPs differences between S and D, we will use the term MMN, while when referring to differences between first‐ and second‐order complexities, which collapse S and D trials, we would use the term N1/MMN.

Four linear mixed‐model analyses (LMM) (JASP 0.19.3.0) were computed on the mean voltage of the ERPs in the selected electrodes and time windows of the CNV, P1, N1/MMN, and PINV components. In order to exploit the possibilities of considering random intercepts and slopes for the within‐subjects fixed factors, an exploratory maximal LMM analysis was computed with type of trial (S and D), complexity (first‐ and second‐order), change of type of trial ([SS and DD] vs. [DS and SD]), electrodes (F3, F4, Fz, C3, C4, and Cz), and participant group (ND and DLD), considering the subjects as a random factor, age and gender as covariates, and each within‐subject factor interacting with the between‐subject factor group. However, in order to permit convergence to estimate intercepts and random slopes, it was necessary to limit random slopes to type of trials and complexity. Additionally, the factor change of type of trial, which did not show any significant effect, was also eliminated from the analysis due to convergence issues. The previous inclusion of the factor change of type of trial was motivated by our previous significant results of this factor with healthy young adults (Ruiz‐Martínez et al. 2022). Therefore, the final LMM model applied to the amplitude of the four ERP components included the within‐subject factors electrode (F3, F4, C3, C4, Cz, Fz), stimulus type (S, D), and stimulus complexity (first‐ and second‐order complexity), each interacting with the between‐subject factor group. Age in days and gender were included as covariates. Random intercepts and random slopes for all within‐subject predictors were specified for each subject to account for inter‐individual variability in baseline voltage and in the effects of the experimental conditions except for the factor electrodes. Interactions of the effects of the participant groups and all other fixed factors were analyzed. Group interactions are the most relevant to test our hypotheses. The R code was exported from JASP and is presented in the supplementary material. In the case that any interaction was significant, additional analyses were computed. These analyses compared the standard vs. deviant trials within each group, or the complexity conditions between both subject groups, depending on whether the interaction with participant group was significant for the trial type or the complexity condition, respectively. For controlling multiple comparisons, the False Discovery Rate was used (FDR) (Benjamini and Hochberg 1995). The reported results would be the fixed‐effects estimates, which also include the direction and magnitude of the effect (parameter *β*).

The ERPs for both types of trials and complexity conditions were displayed for both participant groups across the entire time window from the onset of the S1 to the end of the inter‐stimulus interval, as well as for the 300‐ms post‐S4, in order to better illustrate the effects described in the results.

## 3 Results

**FIGURE 5:**
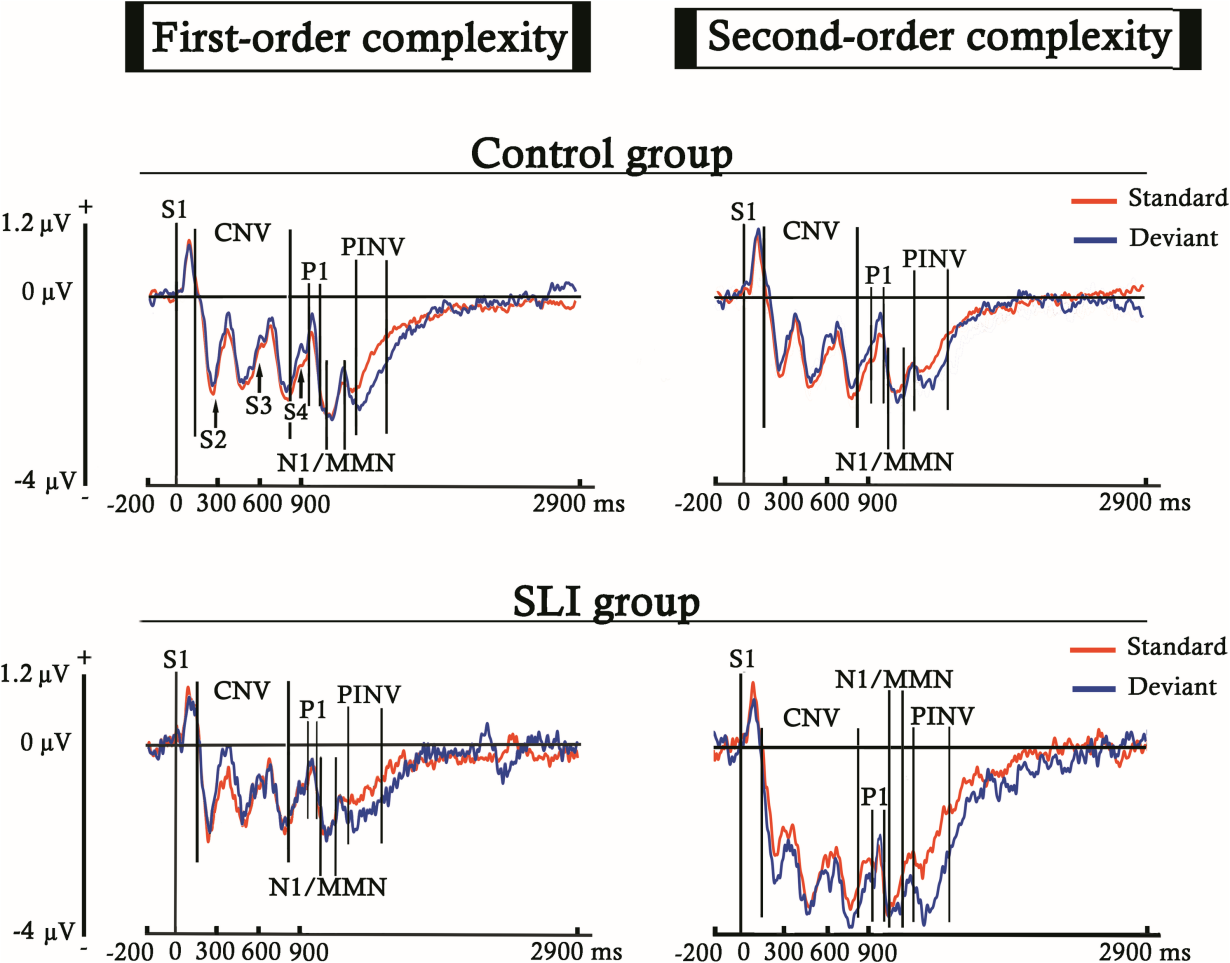
Event‐related potentials (ERPs) elicited by standard and deviant trials, for both the first‐ and second‐order complexity conditions, in the ND and DLD groups. ERPs were obtained by averaging the six electrodes selected for analysis (F3, F4, Fz, C3, C4, Cz) across subjects in each group. The baseline was established before S1 (from −200 to 0 ms), and the plotted time window corresponds to the entire stimulus sequence and the inter‐stimulus interval. The arrows pointing to the waveforms indicate the onset of each stimulus according to its presentation order (Sx). Between bars, are delimited the latencies selected for the components analyzed (CNV, P1, N1/MMN, and PINV). The N1/MMN label indicates that the MMN was embedded within a latency similar to that of the N1 component.

**FIGURE 6:**
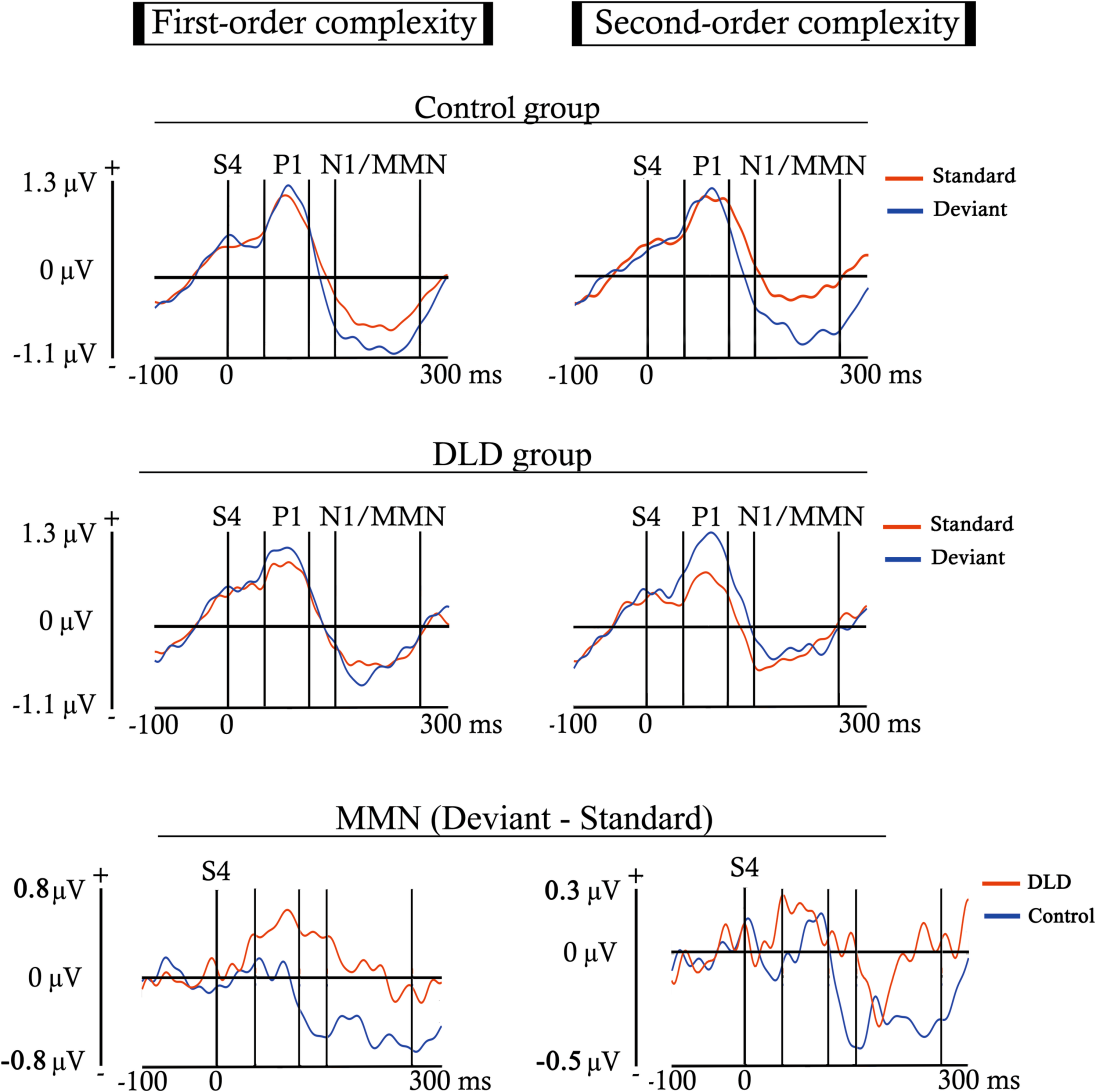
Event‐related potentials (ERPs) elicited by standard and deviant trials, for both the first‐ and second‐order complexity conditions, in the ND and DLD groups. ERPs were obtained by averaging the six electrodes selected for analysis (F3, F4, Fz, C3, C4, Cz) across subjects in each group. The baseline was established before S4 (from −100 to 0 ms), and the plotted time window was 300‐ms post‐S4, allowing for clearer observation of the P1 and MMN components. Between bars, are delimited the latencies selected for the components analyzed (P1 and N1/MMN). The N1/MMN label indicates that the MMN was embedded within a latency similar to that of the N1 component. At the bottom of the figure, the difference wave is presented, obtained by subtracting the standard from the deviant.

After a clearly formed auditory P1, the MMN emerged as a modulation of the N1 component. Both components were observed in the ERP grand‐average (Figures 5 and 6). Additionally, a CNV component was induced during the presentation of the four tones that composed the stimuli sequences (Figure 5). The negativity generated by the CNV is followed by a recovery to the baseline, starting about 350 ms after the S4 onset, which was categorized as a PINV component.

Below, we present the ERPs generated for each trial type, depending on the complexity condition and participant group. Figure 5 shows the ERP waveforms from the onset of the first stimulus of the sequence (S1) to the end of the interstimulus interval. In contrast, Figure 6 displays the same potentials but restricted to the time window post‐S4 selected for the P1 and MMN analyses, so that the effects described in these components can be more clearly observed.

The fixed‐effects estimates of the mixed model analysis (LMM) computed for the P1 component (Figure 6), with baseline located before S4, showed a trend toward significance effect for the interaction between the participant group and the type of trial (S or D) (*β* = 0.27, SE = 0.14, *t* = 1.9, *p* = 0.06). This statistical trend between groups was due to the significant comparison between the S and D trial in the DLD group (*β* = 0.32, SE = 0.12, *t* = 2.7, *p* = 0.018; FDR corrected), caused by the higher response elicited by the D (mean = 1.02 μV ± 2.31 SD) in comparison with the S (mean = 0.7 μV ± 1.68 SD). The increase in P1 amplitude was not significant in the ND group. Additionally, the electrodes analyzed (F3, F4, C3, C4, Cz, and Fz) also presented significant differences between them (*β* = −0.06, SE = 0.16, *t* = −3.78, *p* = 1.6 * 10^−4^).

The analysis computed for the N1/MMN, with baseline located before S4 (Figure 6), showed significant effects for the type of trial and its interaction with the participant groups (Table 4).

**TABLE 4:** Significant fixed‐effects estimates from the LMM results of N1/MMN. The table presents the significant results obtained from the linear mixed model (LMM) analysis of the mismatch negativity (N1/MMN) component.

Effect	*β*	SE	*t*	*p*
Type of trial	−0.82	0.32	−26	0.011
Type of trial * Group	0.42	0.211	1.94	0.055

The difference between the type of trial (S and D) is explained by a higher amplitude of the D trial (mean = −0.63 μV ± 2.37 SD) relative to the S (mean = −0.39 μV ± 1.7 SD). The trend toward significance in the interaction between the participant group and the type of trial is explained by a significant difference shown by the ND group (*β* = −0.41, SE = 0.12, *t* = −3.3, *p* = 0.004; FDR corrected), in contrast to the DLD group. The difference between the type of trial found in the ND group was driven by a higher activation for the D (mean = −0.79 μV ± 2.21 SD) relative to the S (mean = −0.38 μV ± 1.56 SD). These results together suggest that, while the D vs. S comparison in the DLD group is indexed by the P1 component, in the ND group it is indexed by the MMN.

The results obtained for the LMM computed for the PINV component showed significant effects for the complexity condition, as well as for its interaction with the participant group. Moreover, the participant group condition and the age measured in days also showed significant results (Table 5).

**TABLE 5:** Significant fixed‐effects estimates from the LMM results of PINV. The table presents the significant results obtained from the linear mixed model (LMM) analysis of the postimperative negative variation (PINV) component.

Effect	β	SE	t	p
Age in days	−3.87 * 10^−4^	1.94 * 10^−4^	−1.98	0.051
Type of trial * Group	1.89	0.39	4.86	5.521 * 10^−6^
Group	2.41	0.58	−14.14	7.19 * 10^−5^
Complexity condition * Group	−1.65	0.26	−6.31	1.260 * 10^−8^

The main effects observed for the complexity condition and the group resulted from the higher amplitude generated by the second‐order complexity (mean = −2 μV ± 3.13 SD) and the DLD group (mean = −1.93 μV ± 3.49 SD), in comparison with the first‐order condition (mean = −1.56 μV ± 2.57 SD) and the ND group (mean = −1.68 μV ± 2.36 SD), respectively. The significant result obtained for the age measured in days in this component was driven by a progressively increasing negativity associated with increasing participant age (*β* = −0.08, *t* = 5.17, *p* < 0.001).

The interaction between the complexity condition and the participant groups was due to a significant difference between ND participants (*β* = 0.58, SE = 0.28, *t* = 2.06, *p* = 0.042; FDR corrected) and DLD participants (*β* = −1.11, SE = 0.29, *t* = −3.82, *p* = 5 * 10^−4^; FDR corrected) in both the first‐order and second‐order complexity conditions, although in opposite directions across groups. The ND group showed greater activation for the first‐order complexity (mean = −1.79 μV ± 2.43 SD) relative to the DLD participants (mean = −1.56 μV ± 2.29 SD). In contrast, the DLD group exhibited higher activation in the second‐order condition (mean = −2.67 μV ± 3.99 SD), in comparison with the ND group (mean = −1.56 μV ± 2.29 SD). The increased activity observed in the clinical group for the more complex condition may reflect difficulties in processing stimuli, possibly accompanied by a sense of overload.

The analysis performed for the CNV component revealed significant effects for the participant group, and the age in days, as well as the complexity condition and its interaction with the participant group (Table 6).

**TABLE 6:** Significant fixed‐effects estimates from the LMM results of CNV. The table presents the significant results obtained from the linear mixed model (LMM) analysis of the contingent negative variation (CNV) component.

Effect	β	SE	t	p
Age in days	‐3.98 * 10^−4^	1.77 * 10^−4^	−2.25	0.027
Complexity condition	1.58	0.34	4.68	1.096 * 10^−5^
Group	2.2	0.45	‐ 4.84	4.443 * 10^−6^
Complexity condition * Group	−1.59	0.23	−7.04	5.011 * 10^−10^

The significant results obtained for the participant groups was caused by a higher activity elicited in the DLD group (mean = −1.79 μV ± 2.81 SD), compared with the ND group (mean = −1.31 μV ± 1.86 SD). The significant difference between both complexity conditions was caused by a greater response generated by the second‐order condition (mean = −1.84 μV ± 2.49 SD), in comparison with the first‐order (mean = −1.17 μV ± 2.04 SD). Similar to the PINV component, the effect observed for the age (measured in days) in the CNV was due to an increase in negativity associated with increasing age (*β* = −0.06, *t* = −4.19, *p* < 0.001).

On the other hand, the interaction with the participant groups was driven by a significant effect found in the second‐order condition (*β* = −1.29, SE = 0.29, *t* = −4.46, *p* = 5 * 10^−5^; FDR corrected), but not in the first‐order condition. This difference between the participant groups in the most complex condition was due to a greater activity recorded in the DLD group (mean = −2.62 μV ± 3.07 SD) compared with the ND group (mean = −1.33 μV ± 1.86 SD). The results obtained for this component would support the hypothesis that the second‐order complexity condition induces cognitive overload in the DLD participants. This would also be reinforced by the greater overall activation shown by the DLD participants compared with the ND group.

As the difference in the PINV amplitude between the S and D trials was visually evident in both groups and complexity conditions (Figure 5), an ad hoc analysis with the electrodes and trial type as the only factors was conducted. The results indicated a significant effect for the electrodes recorded (*β* = −1.19, SE = 0.22, *t* = −5.39, *p* = 0.008), and also for the type of trial presented (*β* = −0.53, SE = 0.13, *t* = −3.98, *p* = 1.43 * 10^−4^) driven by higher PINV negativity in D (mean = −2.05 μV ± 3.25 SD) relative to S trials (mean = −1.51 μV ± 2.41 SD). These results would suggest a still‐developing predictive processing mechanism in this component, whose effects would be lost in the complete analysis as they are distributed across the other factors.

Finally, all the results presented were included in the Table 7 to facilitate their visualization, and Supplementary Figure 1 presents data variability for P1, N1/MMN, PINV, and CNV. Supplementary Figure 1 illustrates the typically high variability of ERPs in pediatric populations when a broad age range is considered. It should be emphasized that a substantial proportion of this variability is accounted for by the use of LMM statistical analyses.

**TABLE 7:** Summary of key results by component and group. Summary of all results of interest obtained in the study for each component analyzed. The row ND & DLD shows the results observed in both groups, whereas the rows ND and DLD present the results obtained for each group separately, specifically after an interaction between a factor and the group. The upper arrow indicates that negativity increased with age.

	P1	N1/MMN	CNV	PINV
ND & DLD			DLD > ND ↑ Age = ↑ Negativity	D > S^a^ DLD > ND ↑ Age = ↑ Negativity
ND		D > S		> First order
DLD	D > S		> Second order	> Second order

^a^ This result was obtained when computing the LMM only with the factors electrodes and trial type.

## 4 Discussion

The present study, which is grounded in predictive coding theory (Friston 2005; Winkler and Czigler 2012), compared the psychophysiological responses of children diagnosed with DLD and an ND group without neurological disorders while presenting two passive auditory conditions of varying complexity. The aim was to explore whether a deficit or alteration in predictive neural mechanisms could underlie the language development difficulties observed in DLD, as suggested by the statistical learning model proposed for this disorder (Lammertink et al. 2017). The experimental paradigm was designed to stress the predictive mechanisms. Ultimately, the findings may support the development of a child‐friendly protocol to aid in early diagnosis.

As proposed in the first study hypothesis, a stronger psychophysiological response was observed for the deviant (D) compared with the standard (S) trial, reflected in the MMN for the ND group (Table 4) and in the P1 component for the DLD participants (Figure 6). This effect suggests that both groups exhibit predictive processing and subsequent prediction error, regardless of complexity, as they respond more strongly to deviations from the extracted patterns. However, the ND group would show a more typical development of the neural mechanisms involved in prediction, which is supported by the MMN, an index of prediction error processing, as observed in healthy adults and children using the same (Ruiz‐Martínez et al. 2022) or similar experimental designs (Tervaniemi et al. 1994; Muñoz‐Caracuel et al. 2024).

The MMN is more associated with prediction error and deeper cognitive and attentional mechanisms involved in extracting predictive patterns (Winkler 2007; Winkler and Czigler 2012; Paavilainen 2013), which has been related to typical language development (Cheour et al. 2000; Wang et al. 2012; Paquette et al. 2013), or its impairment when the component is reduced or absent (Shafer et al. 2005; Datta et al. 2010; Kujala and Leminen 2017). For this reason, the fact that the DLD participants showed a discrimination effect between S and D trials in the P1 component instead of in the MMN would suggest predictive processing based on more basic sensory‐encoded features of the auditory stimulus (Herrmann and Knight 2001; Pratt 2011). This role of the P1 component in deviance detection is aligned with the idea of a hierarchically organized novelty and deviance detection system in the human auditory system, which would present early indices of deviance detection preceding the MMN, which is more involved in the extraction of abstract regularities (Slabu et al. 2010; Grimm et al. 2011; Grimm and Escera 2012).

Additionally, the fact that predictive processing in DLD was observed in the P1 component, rather than the MMN (Figure 6), may reflect an impairment in the typical development of the brain regions involved in predictive coding. The typical maturation of the central auditory system shows a progressive P1 reduction from childhood (Ponton et al. 2002; Mahajan and McArthur 2012; Sharma et al. 2005; Wunderlich et al. 2006), while the MMN increases into adulthood, in a process in which the neural generators associated with the MMN gradually acquire greater functional relevance (Gomot et al. 2000; Kushnerenko et al. 2002; Guzzetta et al. 2011). Although both components originate in the primary auditory cortex (specifically in Heschl's gyrus) (Fitzroy et al. 2015; Ruhnau et al. 2011), the MMN also involves frontal generators related to attention‐switching or orienting that are triggered by the change detection mechanism (Giard et al. 1990; Rinne et al. 2000; Garrido et al. 2009). In contrast, the frontal activity observed for the P1 (see Figure 4) would result from thalamocortical projections (Ponton and Eggermont 2001). It can be argued that DLD children are able to process the predictive aspect of the signal in P1, indicating a reorganization of the prediction error function. The P1 modulation to deviants, although P1 can be modulated by stimulus‐specific adaptation (SSA) (Ruiz‐Martínez et al. 2022; Cary et al. 2024), cannot be ascribed in the present experiment to SSA, given the low repetition frequency of the fourth tone of the sequence (most extreme in the second‐order condition), and the predictability structure of the experimental paradigm. The P1 modulation in DLD would possibly reflect a functional impairment upstream in auditory processing that is compensated downstream. Thus, the involvement of P1 in predictive processing in children with DLD, instead of the MMN, may reflect a psychophysiological compensation mechanism for an impaired or atypically slow maturation of the frontal and/or auditory MMN generators. However, the obtained amplitude increase in P1 for the DLD group, while MMN is not obtained, and given the broad age range of the present report, would possibly represent different brain processes at different ages, ranging from inefficient processing strategies in preschoolers to a failure to consolidate mature MMN‐based predictive coding in older children.

The presence of a similar amplitude CNV component would suggest that both groups correctly allocated attention and anticipated upcoming stimuli. This effect is consistent with previous findings, showing that CNV amplitude increases as the predictability of a stimulus rises (Barry et al. 2007; Arjona and Gómez 2014; Duma et al. 2020). However, to account for how the previous trial influences the anticipatory response in the current trial, as indexed by CNV, a sequential analysis that considers trial outcomes would be required. Preliminary analyses, as described in the Methods section, revealed no effects of the change/no change factor of the previous‐current trial dyad and did not generate amplitude modulation of the analyzed ERPs of the current trial. This suggests that the passive experimental four sequential tones protocol used in the present study did not induce local sequential effects on ERPs, in contrast to findings from the attentionally active central cue Posner paradigm (Arjona and Gómez 2014). On the other hand, the PINV did not show a significant difference between both types of trials until, motivated by the effect visually observable in Figure 5, an additional ad hoc LMM was computed, including only the electrode and trial type factors. This analysis revealed a significant effect for both factors, caused by a higher response elicited by the D, primarily in frontal areas (Figure 4). These results would suggest the involvement of a still‐developing prediction mechanism for this component, as the effects were not sufficiently robust to withstand the influence of other factors.

The second hypothesis of the study was not confirmed, as neither P1 nor N1/MMN showed differences between the first‐order condition and the second‐order condition. This would suggest that, although the neural mechanisms involved in extracting predictions from varying complexity patterns may differ between children and adults, DLD individuals do not show altered complexity processing in the N1/MMN. In line with this, the MMN sensitivity to complexity has been more frequently reported in the literature (Saarinen et al. 1992; Paavilainen et al., 2013; Paavilainen et al. 2018). The lack of difference in the N1/MMN response between the simple and complex condition in both groups contrasts with the effects described in adults using the same experimental design, in which participants showed a greater activation for the second‐order condition in frontal electrodes and Cz, while the first‐order complexity presented a higher amplitude in C3 and C4 (Ruiz‐Martínez et al. 2022). The similar response for both complexity conditions would reflect that the frontal N1/MMN generators, involved in analyzing changes in complex patterns (Korzyukov et al. 2003; Schröger et al. 2007), are still in a maturation process, given the typically slow development of the frontal areas (Bunge et al. 2002; Ivry and Knight 2002). This result does not agree with the findings described by Gumenyuk et al. (2003), who pointed to the slow maturation of the frontal lobes to explain the lower MMN response elicited in frontal areas in children aged 8–14 by the hard condition of a study consisting of two auditory patterns of different complexity, although the substantial differences in predictability and difficulty of the stimuli between Gumenyuk et al. (2003) and the present report, as well as age differences, would justify the obtained differences.

Addressing the third proposed hypothesis, the effects observed for the comparison between both complexity conditions in the PINV also revealed significant effects in opposite directions between groups (Table 5; Figure 5). While ND subjects showed a higher response for the first‐order condition, the DLD participants showed a greater amplitude for the second‐order condition. The absence of differences between the type of trial in this component, when all the factors analyzed were included, and the increased negativity associated with increasing age could imply an immature development of the update‐predictive functions for the PINV in this age range (from 3 to 11 years old). This proposal would be supported by the findings described in adults applying the same or similar experimental design, in which they showed significant effects both in the comparison between the S and D trials (Muñoz‐Caracuel et al. 2024), and in the first‐ and second‐order conditions (Ruiz‐Martínez et al. 2022).

For these reasons, the PINV observed in the ND group could merely reflect the progressive recovery of the CNV, maintaining the amplitude difference between both complexity conditions previously triggered by the MMN. Meanwhile, in the DLD group, the higher response to the second‐order complexity, along with the absence of adequate predictive error, as indexed by P1 but not by MMN, could reflect cognitive overload. This presumed overload would be caused by the greater abstraction demands of the more complex condition, which lead to difficulties in integrating responses into a symbolic framework and/or a perceived lack of control over an aversive (in this case, excessive) stimulus, as described for this component (Delaunoy et al. 1978; Kathmann et al. 1990; Diener et al. 2009). Then, the increase in CNV amplitude in the second‐order complexity condition in DLD participants (Table 6; Figure 5) would possibly indicate a greater anticipatory effort (Kropp et al. 2001; Kononowicz and Penney 2016; Fattapposta et al. 2024). This result, along with the overall increased activity recorded in this group (similar to the effects described in adults by Plante et al. in 2017), supports the presence of both cognitive overload and enhanced effort to reassess stimulus patterns in children with DLD in response to predictive coding paradigms. An interpretative limitation of the present study, given the broad age range of the sample, concerns the possibility that the increased CNV and PINV amplitudes in the complex condition observed in the DLD group, relative to controls, as well as an increase in the first‐order condition in the PINV for controls, may reflect different cognitive processes at different developmental stages, although resulting in a similar ERP signature. Therefore, rather than reflecting statistical learning difficulties in DLD, the CNV and PINV imbalance between groups could be due to general task difficulty demands, which are more challenging for the DLD group to cope with, rather than to a genuine statistical learning deficit.

Given that cognitive processes mature rapidly at the ages reported here, it is always difficult to ascribe a one‐to‐one relationship between ERPs and cognition; however, relying on fundamental processes such as auditory discrimination gives some confidence that, at least in controls, the analyzed ERPs (P1, MMN, PINV, and CNV) index the sensory and cognitive processes widely accepted in the literature. The observed group differences, if not reflecting immaturity, would at least reflect differential auditory and/or cognitive processing in DLD considered as a group, rather than as individuals—something which is beyond the scope of the present statistical approach.

The present findings provide further evidence for possible statistical learning deficits in children with DLD, consistent with previous reports based on linguistic ERPs, although these effects have not always been explicitly interpreted in terms of statistical learning (Bishop and McArthur 2005; Shafer et al. 2007; An et al. 2022; Shafer et al. 2005; Datta et al. 2010; Kujala and Leminen 2017). For some ERP components, the link to statistical learning is more direct. This is the case for the N400, whose amplitude has been shown to vary as a function of cloze probability (Kutas and Hillyard 1984). Accordingly, delayed or absent N400 responses in individuals with DLD have been interpreted as reflecting a failure to develop semantically driven prediction error signals (Shafer et al. 2005; Sabisch et al. 2006; Haebig et al. 2018). In addition, a reduction in the LPC, which is associated with postlexical reanalysis and semantic integration, has also been reported in DLD (Haebig et al. 2018). Language processing also involves predictive syntactic information, violations of which elicit ERP components such as the Early Left Anterior Negativity (ELAN) and the P600. Impairments in the development of these components in response to unexpected syntactic violations have therefore been interpreted as evidence of deficits in predictive coding mechanisms in DLD (Fonteneau and van der Lely 2008; Royle and Courteau 2014; Purdy et al. 2014; Haebig et al. 2017). The present results extend this line of evidence by suggesting that statistical learning deficits in DLD are not confined to the linguistic domain but also affect more basic learning mechanisms, consistent with findings from procedural learning studies. In Serial Reaction Time (SRT) tasks, where reaction times typically decrease for predictable stimulus sequences relative to random sequences, children with DLD show reduced performance gains for predictable sequences (Gabriel et al. 2013), highlighting impairments in the motor component of procedural sequence learning. Furthermore, individuals with DLD have demonstrated difficulties in artificial grammar learning tasks, which require the acquisition of rule‐based structures in artificial languages, as well as in statistical learning paradigms in which participants implicitly extract regularities from sequential input without explicit instruction (Hsu et al. 2014). Taken together, the present findings suggest that the relationship between DLD and sequence learning is likely to play a crucial role in the acquisition of language rules. However, as suggested above, the ERP imbalance between groups could be due to general task difficulty demands rather than to a genuine statistical learning deficit.

## 5 Conclusions

The current study evaluated the statistical learning model as an explanation for language difficulties in children with DLD, using two passive auditory conditions of varying complexity framed within predictive coding theory. The DLD participants showed an absent MMN and a higher P1 response to D tones, which may indicate immature or impaired development of frontal MMN generators and, possibly, a compensatory mechanism involving earlier auditory processing areas. On the other hand, the increased PINV and CNV amplitudes observed in the DLD participants during the most complex condition could suggest cognitive overload and heightened effort to reassess stimulus patterns in this group. Finally, all these findings (see Table 7) would support the statistical learning model as a valid approach for understanding the neural basis of DLD. This could contribute to the development of an early detection protocol through EEG, given its noninvasive, child‐friendly, and passive features.

## 6 Limitations

Between the limitations of the present report, the variety of diagnostic measures precludes the possibility of establishing correlational relationships between EEG and language performance.

## Author Contributions

**Francisco J. Ruiz‐Martínez:** writing – review and editing, writing – original draft, visualization, validation, methodology, investigation, formal analysis, data curation, conceptualization. **Elena I. Rodríguez Martínez:** investigation. **Brenda Angulo Ruiz:** investigation. **Ana Gómez Trevióo:** resources, project administration, investigation, conceptualization. **Raquel Muñoz Pradas:** investigation. **Sheyla Andalia:** methodology. **Carlos M. Gómez:** writing – review and editing, writing – original draft, supervision, validation, resources, project administration, methodology, investigation, funding acquisition, formal analysis, conceptualization.

## Funding

This research was funded by Agencia Estatal de Investigación, grant number PID2022‐139151OB‐I00.

## Conflicts of Interest

The authors declare no conflicts of interest.

## Supporting information


**Figure S1:** Mean and Standard Error of each of the analysed component (P1, N1/MMN, PINV and CNV) for deviants (D) and Standards(S), first order complexity (1st‐O) and second order (2nd‐O) complexity for both groups: Normodevelopment (ND) and Developmental Language Disorder (DLD). Bars correspond to standard errors.

## Data Availability

The data supporting the findings of this study are available at https://github.com/Martinez‐Ruiz/Prediction‐in‐Developmental‐Language‐Disorder‐Data.git.
