# Supplementary material for: Modulation of Predictive Coding in Auditory Paradigms of Varying Complexity in Children With Developmental Language Disorder
Source: Eur J Neurosci. 2026 Apr 24;63(8):e70503. doi: 10.1111/ejn.70503 (PMC13108555; doi:10.1111/ejn.70503)
Supplement: Supplementary file 1 — Figure S1: Mean and Standard Error of each of the analysed component (P1, N1/MMN, PINV and CNV) for deviants (D) and Standards(S), first order complexity (1st‐O) and second order (2nd‐O) complexity for both groups: Normodevelopment (ND) and Developmental Language Disorder (DLD). Bars correspond to standard errors. [file EJN-63-0-s001.pdf]

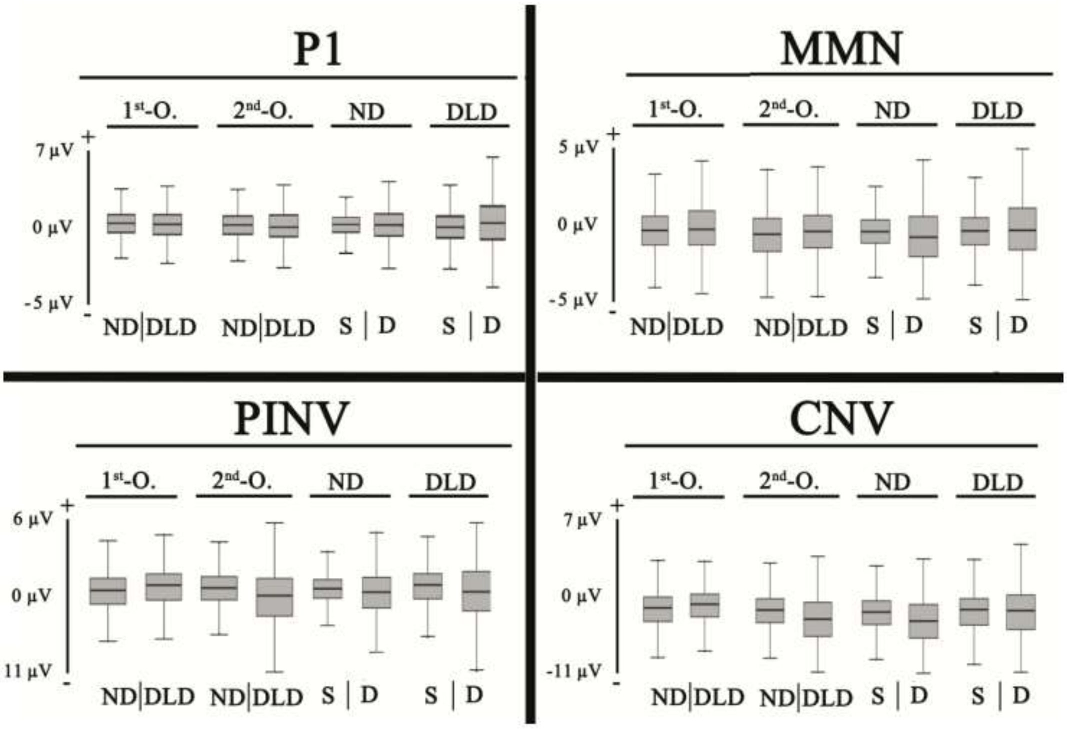

Supplementary Figure 1. Mean and Standard Error of each of the analysed component (P1, N1/MMN, PINV and CNV) for deviants (D) and Standards(S), first order complexity (1st-O) and second order (2nd-O) complexity for both groups: Normodevelopment (ND) and Developmental Language Disorder (DLD). Bars correspond to standard errors.

R syntax:

```
jaspMixedModels::MixedModelsLMM(
    version = "0.19.2",
    formula = voltaje ~ grupo * `F3 F4 C3 C4 Cz Fz` + simple_complejo * grupo + S_D *
grupo + Edad_en_dias + genero + (1 + simple_complejo + S_D | sujeto),
    contrasts = NULL,
    fixedEffectEstimate = TRUE,
    marginalMeansTerms = ~ S_D + grupo,
    modelSummary = TRUE,
    trendsContrasts = NULL)
```

P1 results:

Fixed Effects Estimates

Term	Estimate	SE	df	t	p
Intercept	1.581	0.650	140.797	2.434	0.016
edad_en_dias	-9.798×10^{-5}	9.622×10^{-5}	83.354	-1.018	0.311
genero	0.102	0.123	82.464	0.834	0.406
grupo	-0.416	0.355	86.510	-1.173	0.244
S_D	-0.216	0.213	84.092	-1.015	0.313
simple_complejo	0.043	0.274	83.681	0.157	0.876
F3 F4 C3 C4 Cz Fz	-0.060	0.016	3846.628	-3.778	1.607×10^{-4}
grupo ✻ S_D	0.272	0.143	84.460	1.906	0.060
grupo ✻ simple_complejo	-0.040	0.184	84.074	-0.219	0.827

Note. The intercept corresponds to the (unweighted) grand mean; for each factor with k levels, k - 1 parameters are estimated with sum contrast coding. Consequently, the estimates cannot be directly mapped to factor levels. Use estimated marginal means for obtaining estimates for each factor level/design cell or their differences.

N1/MMN results:

Fixed Effects Estimates

Term	Estimate	SE	df	t	p
Intercept	0.292	0.817	152.795	0.357	0.721
Edad_en_dias	-1.172×10^{-4}	1.159×10^{-4}	82.553	-1.011	0.315
genero	-0.020	0.150	81.764	-0.133	0.895
S_D	-0.824	0.318	84.140	-2.593	0.011
grupo	-0.471	0.457	100.155	-1.032	0.305
simple_complejo	0.424	0.296	82.844	1.431	0.156
F3 F4 C3 C4 Cz Fz	-0.022	0.052	3845.240	-0.417	0.677
S_D ✻ grupo	0.415	0.213	84.427	1.945	0.055
grupo ✻ simple_complejo	-0.140	0.200	83.201	-0.703	0.484
grupo ✻ F3 F4 C3 C4 Cz Fz	0.059	0.035	3845.240	1.669	0.095

Note. The intercept corresponds to the (unweighted) grand mean; for each factor with k levels, k - 1 parameters are estimated with sum contrast coding. Consequently, the estimates cannot be directly mapped to factor levels. Use estimated marginal means for obtaining estimates for each factor level/design cell or their differences.

PINV results:

Fixed Effects Estimates

Term	Estimate	SE	df	t	p
Intercept	-3.291	1.157	151.443	-2.845	0.005
Edad_en_dias	-3.866×10^{-4}	1.948×10^{-4}	83.077	-1.984	0.051
genero	0.249	0.252	82.582	0.989	0.325
simple_complejo	1.888	0.389	83.435	4.856	5.521×10^{-6}
grupo	2.412	0.583	104.002	4.136	7.186×10^{-5}
S_D	-0.361	0.405	82.779	-0.890	0.376
F3 F4 C3 C4 Cz Fz	0.065	0.070	3844.107	0.939	0.348
simple_complejo ✻ grupo	-1.653	0.262	83.649	-6.312	1.260×10^{-8}
grupo ✻ S_D	-0.121	0.272	83.073	-0.446	0.657
grupo ✻ F3 F4 C3 C4 Cz Fz	-0.002	0.047	3844.107	-0.035	0.972

Note. The intercept corresponds to the (unweighted) grand mean; for each factor with k levels, k - 1 parameters are estimated with sum contrast coding. Consequently, the estimates cannot be directly mapped to factor levels. Use estimated marginal means for obtaining estimates for each factor level/design cell or their differences.

CNV results:

Fixed Effects Estimates

Term	Estimate	SE	df	t	p
Intercept	-3.140	0.972	139.058	-3.229	0.002
Edad_en_dias	-3.997×10^{-4}	1.773×10^{-4}	82.208	-2.254	0.027
genero	0.090	0.230	82.116	0.391	0.697
simple_complejo	1.576	0.337	83.110	4.681	1.096×10^{-5}
grupo	2.202	0.455	104.164	4.844	4.443×10^{-6}
S_D	0.454	0.284	84.078	1.598	0.114
F3 F4 C3 C4 Cz Fz	0.079	0.053	3845.382	1.493	0.136
simple_complejo ✻ grupo	-1.598	0.227	83.164	-7.042	5.011×10^{-10}
grupo ✻ S_D	-0.226	0.191	84.402	-1.187	0.239
grupo ✻ F3 F4 C3 C4 Cz Fz	-0.005	0.036	3845.382	-0.130	0.896

Note. The intercept corresponds to the (unweighted) grand mean; for each factor with k levels, k - 1 parameters are estimated with sum contrast coding. Consequently, the estimates cannot be directly mapped to factor levels. Use estimated marginal means for obtaining estimates for each factor level/design cell or their differences.
